# Resolution-enhanced imaging using interferenceless coded aperture correlation holography with sparse point response

**DOI:** 10.1038/s41598-020-61754-0

**Published:** 2020-03-19

**Authors:** Mani Ratnam Rai, Joseph Rosen

**Affiliations:** School of Electrical and Computer Engineering, Ben-Gurion University of the Negev, P.O. Box 653, Beer-Sheva, 8410501 Israel

**Keywords:** Optics and photonics, Imaging and sensing

## Abstract

Interferenceless coded aperture correlation holography (I-COACH) is a non-scanning, motionless, incoherent digital holography technique. In this study we use a special type of I-COACH in which its point spread hologram (PSH) is ensemble of sparse dots. With this PSH an imaging resolution beyond the classic diffraction limit is demonstrated. This resolution improvement is achieved due to the position of the coded aperture between the object and the lens-based imaging system. The coded aperture scatters part of the light, that otherwise is blocked by the system aperture, into the optical system, and by doing that, extends the effective numerical aperture of the system. The use of sparse PSH increases the signal-to-noise ratio of the entire imaging system. A lateral resolution enhancement by a factor of about 1.6 was noted in the case of I-COACH compared to direct imaging.

## Introduction

The quest for innovations in imaging with an improved lateral resolution has been carried out for a long time^1–3^. The main challenge of improving the resolution by the various techniques of superresolution is to detour the diffraction limit without reducing the wavelength of the illuminating light and without increasing the aperture diameter of the optical system^4,5^. The entire resolution improvement techniques can be roughly sorted to those that improve the image resolution with the same amount of object information, in contrast to those methods that increase the amount of information introduced into the system with the same physical aperture. Object information in this context means the spatial spectrum of the object’s image. Spatial filtering^6^, digital post-processing^7^ and engineering the point spread function^8^ are a few examples of resolution enhancement with the same spatial bandwidth used by the original systems. In general, the methods based on the same spatial bandwidth have been found less effective in a practical noisy environment. More noise-immune examples of this group are the incoherent digital holography techniques such as Fresnel incoherent correlation holography (FINCH)^9^, and Fourier incoherent single channel holography (FISCH)^10^. Improved image resolution has been observed in FINCH and FISCH systems in comparison to conventional imaging systems and this improvement has been achieved without increasing the numerical aperture (NA) or the bandwidth of the systems.

The other group of methods in which the captured bandwidth is extended has shown more successes in general. Structured illumination^11,12^ is an example in which more object information is introduced by projecting gratings on the object. Effectively, the NA and the spatial bandwidth are increased without changing the aperture size. Synthetic aperture^13,14^ is another example in which the system covers a virtual aperture along the time and by that, extends the effective size of the aperture without actually changing the physical aperture. Other examples are fluorescence imaging techniques such as stochastic optical reconstruction microscopy (STORM)^15^ and stimulated emission depletion microscopy (STED)^16^. In general, more information is grabbed by spending other system resources such as the capturing time. In other words, the time resolution is reduced in order to improve the spatial resolution.

Lately, a new technique named, interferenceless coded aperture correlation holography (I-COACH)^17^ has been developed which has imaging features better than of regular imaging in some aspects. Moreover, I-COACH has 3D imaging capabilities which can be done using three^18^, or two^19^ camera shots, or even a single shot^20,21^. In I-COACH, light radiated form the object is modulated by coded random phase mask (CPM), propagates in the free space and recorded by a digital camera as an object hologram. The object reconstruction is done by correlating the object hologram with a library of point spread holograms (PSHs), where each PSH is recorded for a point object placed at different axial location of the imaging system. I-CAOCH was further developed for extending the system’s field of view^22^, for imaging through partial apertures^23^, for better resolving image details^8,24,25^ and for seeing through scattering layers^26^. Due to the lack of two wave interference in the I-COACH holography, the optical configurations and calibrations are simpler in comparison to traditional holographic systems.

Following the invention of the basic I-COACH, our group developed a version with the properties of improved resolution^25^. Although this method introduces more information into the system, and although the effective NA and the spatial bandwidth are increased, the technique uses only a single camera shot using the method of nonlinear correlation^21,26^. In other words, the resolution improvement in I-COACH is unique in the sense that it is achieved without scarifying the time resolution.

Another recent development in this field of incoherent digital holography is the sparse I-COACH (SI-COACH)^27^. In SI-COACH the CPM is engineered such that the PSH is ensemble of dots sparsely distributed over the hologram plane. This appearance of the dots is the midway between the single dot of the conventional direct imaging and the continuous chaotic distribution of the early versions of I-COACH. As a midway response, it has some of the properties of both imaging concepts. On one hand, because the light from the source point split to relatively low number of isolated image points, the signal-to-noise ratio (SNR) of the recoded hologram is higher than in the old version of I-COACH. On the other hand, the sparse PSH is more complicated pattern than the single dot and hence the background noise of the reconstructed image is tolerable. The chosen dot density is determined as the density which yields maximum SNR and minimum background noise.

In this study we continue developing the technique of resolution enhancement by integrating the SI-COACH^27^ into the method of the single-shot resolution enhancement^25^. Since the use of SI-COACH increases the SNR, we are capable of imaging reflective objects. Hence, the four elements in this study, which have not appeared together in any previous publication, are: 1. The use of sparse PSHs in SI-COACH. 2. Imaging of weakly illuminated reflective (and transmission) objects. 3. Imaging of a scene containing multiple axial planes. 4. The image reconstruction is carried out by a nonlinear correlation. Although each of these elements has been published separately by our group, these four elements are for the first time integrated together for demonstrating resolution-enhanced imaging. The new method in this study is called sparse resolution improved COACH (SRI-COACH).

## Results

### 2D resolution-improved imaging

**Figure 1:**
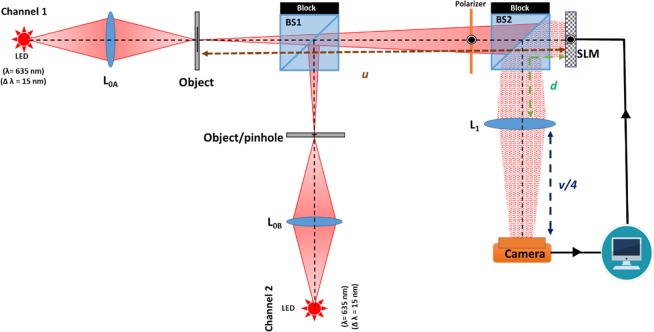
Experimental setup of the interferenceless coded aperture correlation holography with sparse resolution improved COACH (SRI-COACH). BS1 and BS2 – Beam splitters; SLM – Spatial light modulator; *L*_0A_, *L*_0B_ and *L*_1_ – Refractive lenses; LED– Light emitting diodes; P- Polarizer.

The experimental setup used for the verification of the proposed technique is shown in Fig. 1. LEDs (Thorlabs LED635L, *λ* = 635 *nm*, Δ*λ* = 15 *nm*, 170 *mW*) in combination with lenses (*L*_*0A*_ and *L*_*0B*_) were used to critically illuminate^17^ the objects and a pinhole. Critically illumination means that the wide spatially incoherent light source is projected on the object to guarantee full incoherence between any two points on the object. In channel 1, a pinhole of size 25 *μm* is used to record the PSH on the camera plane, whereas in channel 2, elements 1–6 in Group 3 of United States Air Force (USAF) resolution transmission target were used as the object. The object and the pinhole were kept at a distance of 49 *cm* from the phase-only reflective spatial light modulator (SLM) (Holoeye PLUTO, phase-only modulation, 1920 × 1080 pixels, 8 *μm* pixel pitch). The lens *L*_1_ of 30* cm* focal length was kept at a distance of 11* cm* from the SLM. An iris of diameter *D* = 4* mm* was mounted in front of lens *L*_1_ to control the initial NA of the lens-based system. The distance between the lens *L*_1_ and the digital camera (Retiga-R6, 2688 × 2200 pixels, 4.54 *μm* pixel pitch, monochrome) was 15* cm*.

**Figure 2:**
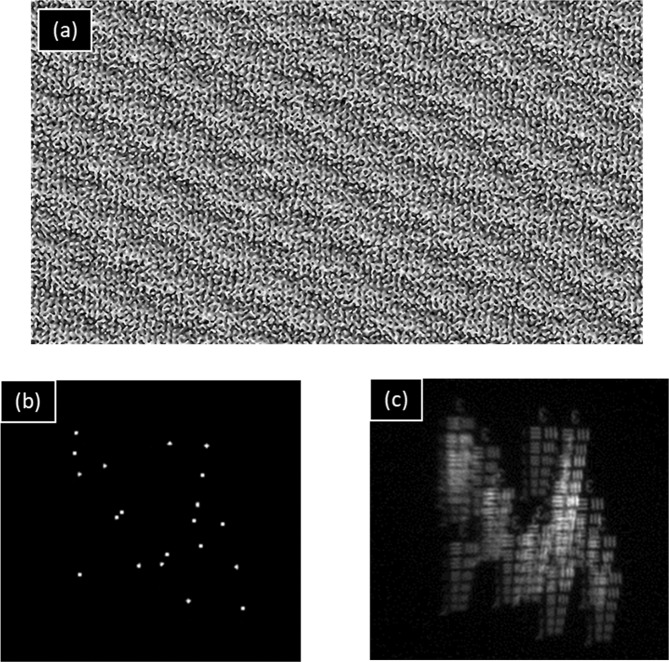
(**a**) Image of 1920 × 1080 pixels of the coded phase mask (CPM) generated using the Gerchberg-Saxton algorithm (GSA), (**b**) intensity of point spread hologram (PSH) for 25 *μm* pinhole and (**c**) object intensity responses of USAF target Group 3 for scattering degree (*σ*_*o*_) = 0.132 and dot number (*N*_o_) = 20.

The phase pattern on the SLM is generated by the modulo-2π phase addition of the CPM with the diffractive lens (DL) of a 23.3 *cm* focal length. Gerchberg-Saxton algorithm (GSA) (to be described in Materials and Methods) is used to generate the CPM. The Fourier transform relations of the GSA between the SLM and sensor plane is experimentally satisfied by combining the DL with CPM. CPMs with different scattering degrees are generated by varying the area constraint in the spectral domain, whereas the scattering degree of CPM (*σ*) is defined as the ratio between the constrained and the maximal possible spectral bandwidths. In other words, the scattering degree is directly controlled in the GSA by the constraint area on the sensor plane, and *σ* is maximal, and equal to 1, when the GSA lets the light to scatter over the entire sensor area. In the experiment, the scattering degree (*σ*) was varied from 0.052 to 0.39 with the step size of 0.026, whereas the number of dots for each scattering degree is varied in the range of *N* = 10–10,000. CPM without the DL, PSH, and object response for the scattering degree 0.132 and 20 dots are shown in Fig. 2.

**Figure 3:**
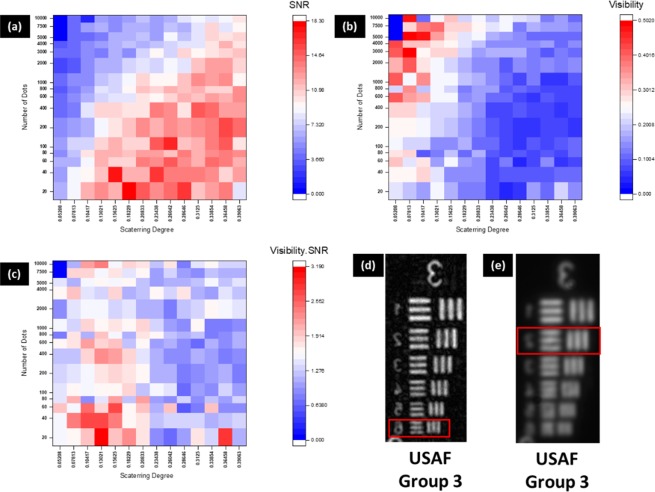
(**a**) Signal to Noise Ratio (SNR), (**b**) Visibility, (**c**) $\xi (N,\sigma ),$ (**d**) Reconstructed image of SRI-COACH for optimal dot number (*N*_o_) = 20 and optimal scattering degree (*σ*_*o*_) = 0.132 and (**e**) Direct imaging. (Red boxes indicate the gratings with minimal resolvable cycles).

The plots of SNR, visibility and $\xi (N,\sigma )$ (The product Visibility × SNR) for USAF target (Group 3 Element 6), with varying scattering degree and dot number are shown in Fig. 3(a–c). As shown in Fig. 3(b) the maximum visibility of 0.502 is recorded for *σ* = 0.078 and *N* = 10,000. In case of SNR, as shown in Fig. 3(a), the maximum value of 18.3 is recorded for *σ* = 0.156 and *N* = 40. The optimal scattering degree (*σ*_*o*_) and optimal dot number (*N*_o_) are those in which $\xi (N,\sigma )$*,* shown in Fig. 3(c), is maximal. The highest value of $\xi (N,\sigma )$ is 3.19 obtained for *N*_o_ = 20 and *σ*_*o*_ = 0.132. The object reconstruction for optimal scattering degree and dot numbers is shown in Fig. 3(d) and is compared with the direct imaging (DI) seen in Fig. 3(e). The minimum feature resolvable for SRI-COACH is 14.25 *lp/mm*, whereas for DI is 8.98 *lp/mm* resulting in improvement of the resolution by a factor of about 1.6.

### Multiplane resolution-enhanced imaging

**Figure 4:**
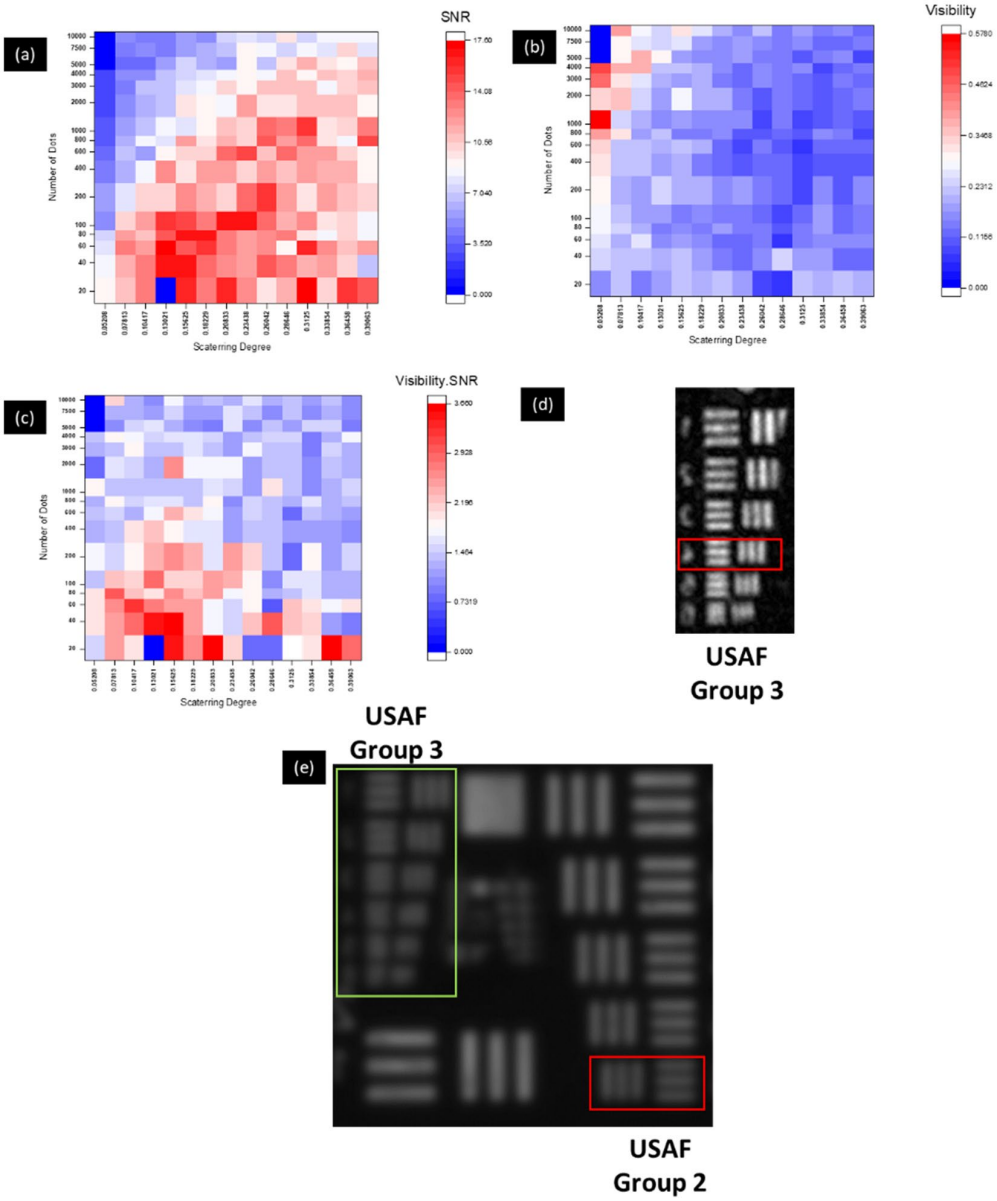
(**a**) Signal to Noise Ratio (SNR), (**b**) Visibility, (**c**) $\xi (N,\sigma )$,(**d**) Reconstructed image of SRI-COACH for optimal dot number (*N*_o_) = 40 and optimal scattering degree (*σ*_*o*_) = 0.156 and (**e**) Direct imaging (Green box represents the USAF group used for SRI-COACH. Red boxes indicate the gratings with minimal resolvable cycles).

In the multiplane experiments, resolution-improved imaging was carried out for two objects, where each of the objects was mounted in each of the two channels at different axial location. Plane 1 was at the same axial location as in the previous section. Hence, all the experimental parameters are the same and the CPM parameters are *N*_o_ = 20 and *σ*_*o*_ = 0.132. For the plane 2, the second object was positioned at a distance of 64 *cm* from the SLM and the DL displaced on the SLM was with a focal length of 26.6 *cm*. Since the optimal scattering degree and sparsity varies with the axial location, plane 2 was calibrated for its optimal values. SNR, visibility and $\xi (N,\sigma )$ values for USAF target (Group 3 Element 4) of SRI-COACH for different dot number and scattering degree are shown in Fig. 4(a–c) for plane 2. An optimal reconstruction was achieved for *N*_o_ = 40 and *σ*_*o*_ = 0.156 and resolution enhancement of about 1.6 {minimum resolved grating for SRI-COACH is shown in red box in Fig. 4(d) and for DI [Fig. 4(e)], whereas the minimal resolvable element is 6 of group 2 with 7.13 lp/mm}.

**Figure 5:**
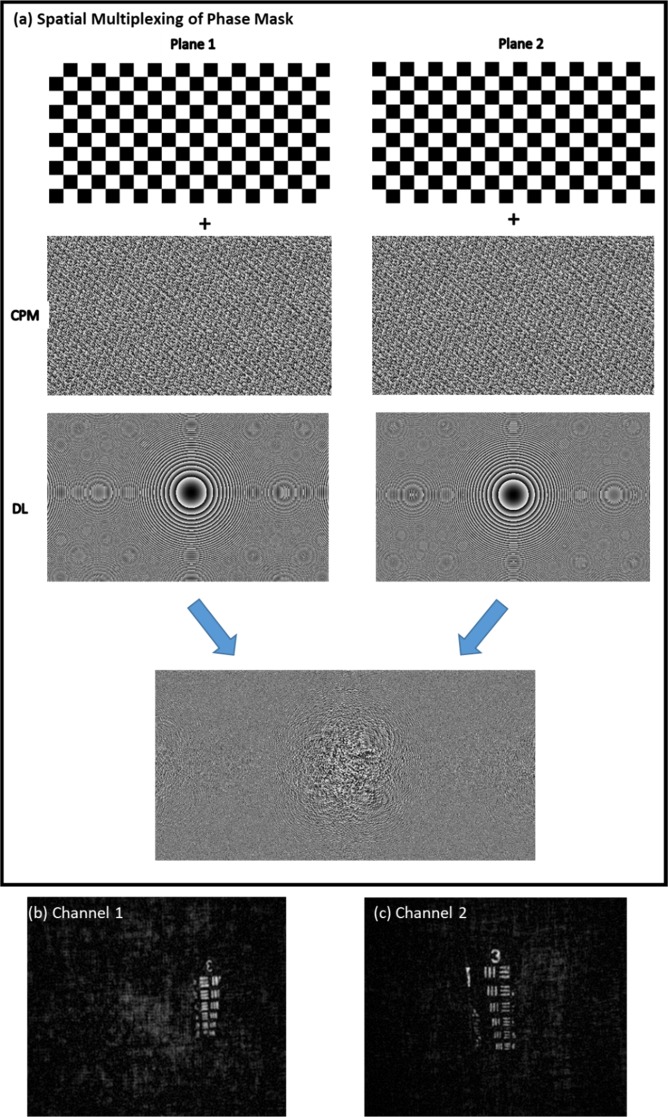
(**a**) Checkerboard-based multiplexing process for final phase mask generation to enable multiplane imaging and reconstruction results of (**b**) plane 1, (**c**) plane 2 separated by a distance of 15 *cm*.

As mentioned above, plane 1 and 2 have different values of *N*_o_ and *σ*_*o*_ and both planes require different DL to satisfy the imaging condition from object to camera plane. As discussed in the following, the imaging condition between object and camera plane is necessary for a proper CPM synthesis. In order to have multiplane imaging, CPM and DL for each plane should be multiplexed on the SLM. In the case of two-plane imaging, the final display pattern for each plane was multiplexed in a checkerboard order as shown in Fig. 5(a). One CPM with its corresponding DL for plane 1 is displayed on even squares and the other CPM with its DL for plane 2 on odd squares, where each square consists of 10 pixels of the SLM. Two PSHs were prerecorded for a pinhole of 25 *μm*, for each axial location and for each corresponding CPM. Object reconstruction for different axial location is done by nonlinear correlation^21^ of object hologram with the corresponding axial PSH. In each channel the USAF target (Group 3) is chosen as the object and the reconstructed images for plane 1 and 2 are shown in Fig. 5(b–c).

### SRI-CAOCH for reflective objects

**Figure 6:**
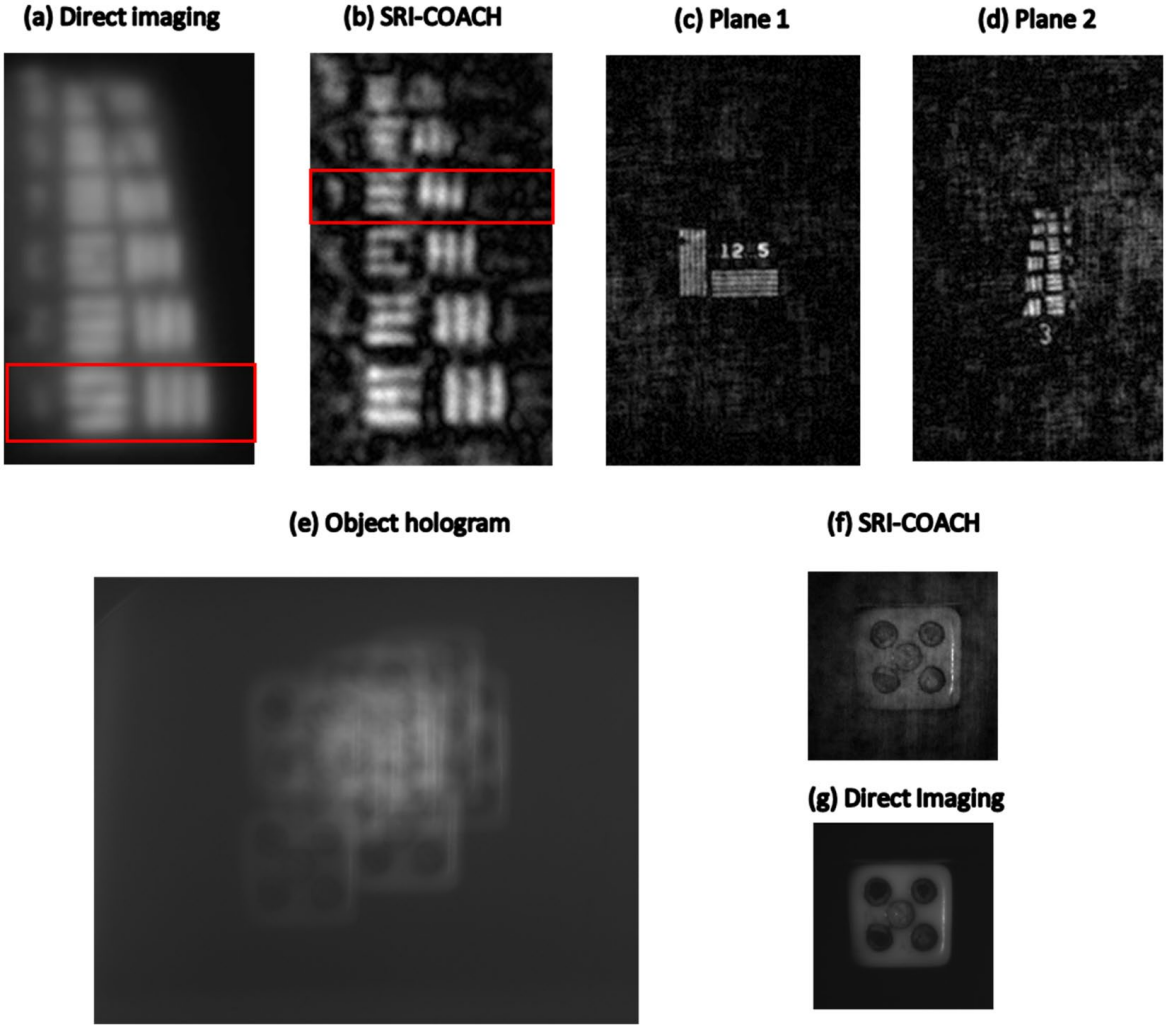
Imaging results of various objects all illuminated in reflective mode. (**a**) Direct imaging and (**b**) reconstruction results of SRI-COACH for minimum resolvable features in the USAF target (Group 3). Multiplane SRI-COACH results for (**c**) plane 1 (NBS target (12.5lp//mm)), (**d**) plane 2 (USAF target (Group 3)) separated by a distance of 15 *cm*. (**e**) Object hologram (SRI-COACH), (**f**) reconstructed image by SRI-COACH and (**g**) direct imaging for a grey-scale object. (Red boxes indicate the gratings with minimal resolvable cycles).

Similar experiments were repeated for the USAF negative (reflective) target (Group 3). The experimental setup is the same as in Fig. 1, but instead of illuminating the targets in a transmission mode they were illuminated such that the reflective light was grabbed into, and processed by, the system. Two sets of experiments were carried out for reflective objects. The first experiment demonstrates the resolution improvement and the second shows the multi-plane imaging capability. Object and pinhole (25 *μm*) were positioned at a distance of 49 *cm* from the SLM and the rest of the experimental parameters were kept the same as described in the previous sections. CPM corresponding to *N*_o_ = 20 and *σ*_*o*_ = 0.132 in combination with DL of focal length of 23.3 *cm* was displayed on the SLM. Reconstruction result for direct imaging and SRI-COACH result are shown in Fig. 6(a,b). Minimum feature resolvable for SRI-COACH is 11.31 *Lp/mm* whereas for DI is 8 *lp/mm* marked inside the red box, resulting in a resolution enhancement by about a factor of 1.4.

In the second set of experiments, the multiplane reconstruction of the proposed method with the reflective object was demonstrated. Experiential parameters, axial location, CPMs and DLs of these experiments were kept the same as that of the previous sections. Phase mask displayed on SLM is constructed by checkerboard multiplexing technique for the two-plane experiment. The tested objects were USAF target (Group 3 & Element 1–6) and NBS target (Element 12.5 *lp/mm*). Pinhole of 25 *μm* was used for recording PSHs. Object reconstructions for different plane are shown in Fig. 6(c,d).

In the last set of experiments, a dice as a real-world object was tested in order to examine the method capability in imaging grey-level objects. The semi diffusive surface of the dice creates a grey-scale object in contrast to USAF target which is characterized as a binary image. This set of experiments demonstrates the imaging capability of SRI-COACH for grey-scale realistic objects. The object was kept at a distance of 49 cm from the SLM and DL of 23.3* cm* focal length in combination with CPM corresponding to *N*_o_ = 20 and *σ*_*o*_ = 0.132 was displayed on the SLM. The object hologram, reconstructed image from SRI-COACH and direct imaging result are shown in Fig. 6(e-g), respectively.

## Discussion

We have demonstrated a new technique for achieving a resolution beyond the diffraction limit in a modified I-COACH system, by enhancing the effective NA and introducing sparsity into the system point response. Effective NA is increased by inserting the SLM in front of the input aperture and displaying a scattering mask to scatter some of the marginal light rays into the optical system. Consequently, the system NA is effectively increased without changing anything in the original aperture. The amount of scattering needed for the resolution enhancement is controlled by the scattering degree of the CPM, generated using the GSA. In our previous work^25^, although the resolution has been enhanced, using scattering masks increased the power losses and consequently relatively low SNR in the reconstructed images has been obtained. Low SNR of the reconstructed images might reduce the resolution to be below the level that can be achieved without the proposed method. In order to improve the power efficiency of the captured holograms, the GSA is modified to generate PSH of sparse dots rather than the continuous intensity on the camera plane as proposed previously. A pattern of randomly distributed sparse dots is the compromise between a single dot of the DI with maximum power efficiency but with a conventional diffraction-limited resolution, and the continuous intensity PSH with the improved resolution, but also with relatively low power efficiency.

GSA used in SRI-COACH has two parameters. First the scattering degree which is varied from 0.052 to 0.39 to control the scattering, and the other parameter is the number of dots on the camera plane varying from 10 to 10,000 to control the sparsity. To determine the optimal scattering degree and the sparsity, the visibility and SNR of the reconstructed image have been examined. The product of visibility and SNR values are considered as the figure of merit, and the combination of scattering degree and sparsity that gives the highest value was chosen as the optimal pair for resolution enhancement. The gain in power efficiency and SNR enable us to implement multiplane imaging by multiplexing two CPMs in a checkerboard arrangement^27^ and enable us to image objects in a reflective mode.

SRI-COACH has shown improvement of the resolution by a factor of about 1.6 for transmission objects and 1.4 for the reflective objects, in comparison to the direct imaging. The proposed technique has demonstrated the resolution improvement using a scattering mask without the drawback of too much power loss resulting from the scattering. As before^25^ the SNR and the resolution enhancement can be traded off against each other by changing the scattering degree. In addition, the SRI-COACH has been demonstrated for imaging a grey-scale, real-world object. In a qualitative comparison to direct imaging result [Fig. 6(g)], the image edges are sharper and the visibility of the details is higher in the SRI-COACH image [Fig. 6(f)].

The proposed technique is universal in the sense that it is not limited to only a single lens optical system, or to any other specific optical imager. Actually, the method can be used with any imaging system such as microscopes, telescopes and almost any other diffraction limited system. In principle, the original system can remain in the same configuration besides the CPM that should be introduced in the space between the object and system entrance. We realize that in the present experiment we do not keep the non-modification principle by shifting the camera toward the refractive lens a distance of 75% of the original gap of camera-lens (See the next section of Materials and Methods). This modification is necessary only because of the non-ideal operation of the SLM in which high percentage of the light is reflected unmodulated. Using more advanced SLMs with negligible unmodulated light can enable implementing the SRI-COACH in various optical systems without changing their original configurations. Although the current method is limited to spatially incoherent illumination, a similar coherently illuminated system can, and probably soon will, be implemented based on the recently proposed method of coherent I-COACH^28^.

The interferenceless and motionless characteristic with an optical configuration as simple as a regular lens-based imaging system makes SRI-COACH advantageous over other conventional resolution-enhanced techniques. The single shot approach and the lack of complex computational procedures yield a method of resolution improvement without any loss of the time resolution.

## Materials and Methods

### General scheme

**Figure 7:**
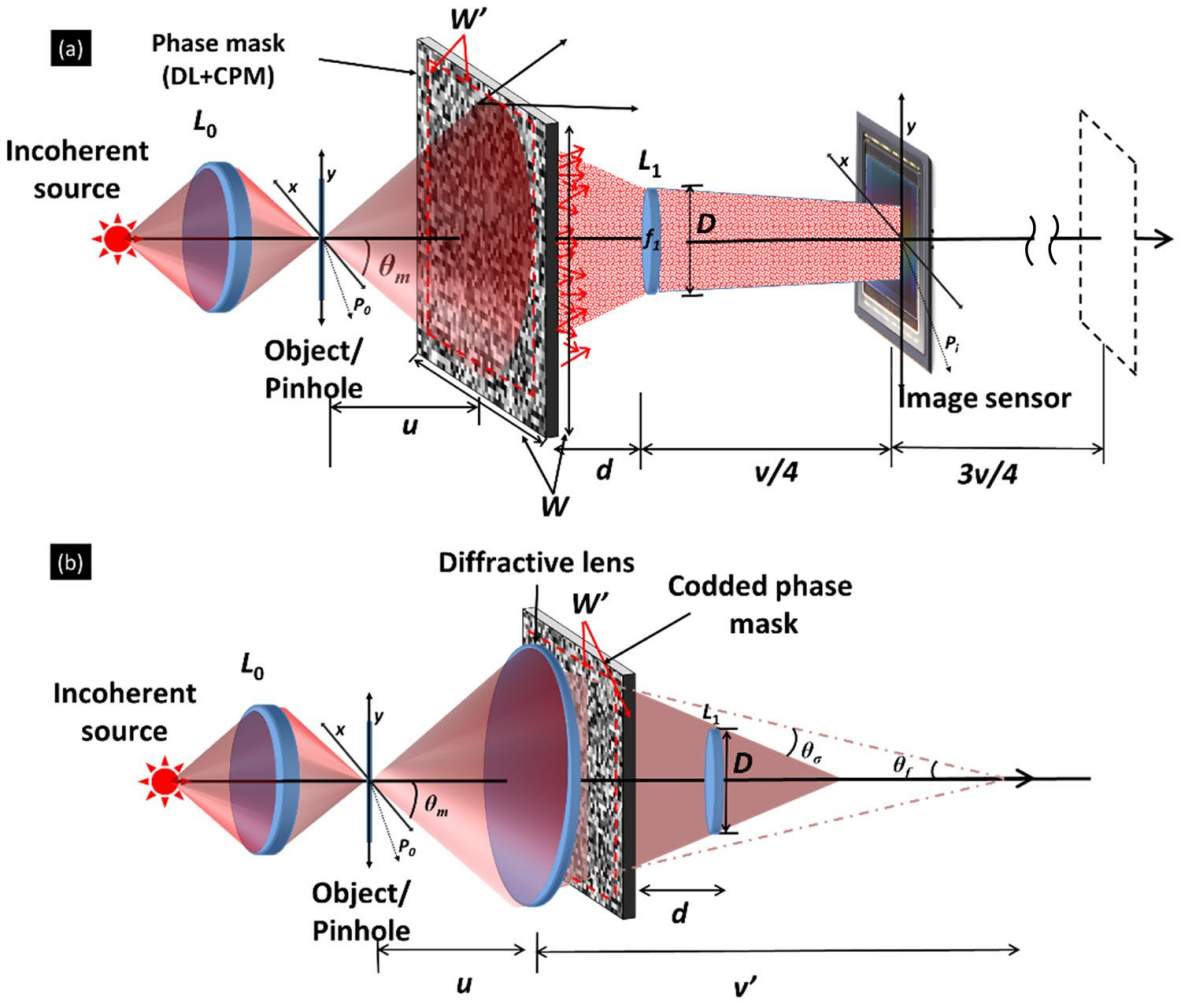
(**a**) Optical scheme of the sparse resolution improved coded aperture correlation holography (SRI-COACH) system. *L*_0_ and *L*_1_ are refractive lenses; *f*_1_ is the focal length of lens *L*_1_; Broken lines indicate direct imaging mode and (**b**) Optical ray schematic describing the effective increment of the numerical aperture by the combination of diffractive lens and coded phase mask.

The schematic configuration of the experimental setup is shown in Fig. 7. Light from an incoherent source critically illuminates an object using a refractive lens *L*_0_. The light diffracted from the object is modulated by the phase mask displayed on the SLM. The phase mask consists of the CPM and the DL, where the CPM is synthesized using the modified GSA. The light modulated by the SLM is collected by lens *L*_1_ and focused on the camera. The camera location is different between two configurations in this work. In the first case of DI, no phase mask is displayed on the SLM and the aim is to determine the inherent resolution of the imaging system. For this case the camera is designed to satisfy the imaging condition between the object and camera where *L*_1_ is the only imaging lens. In the second case of SRI-COACH, the camera is positioned at the quarter distance of the DI camera-SLM gap. There are two reasons to choose this distance. First, we want to minimize the intensity of the unmodulated light reflected from the SLM and focused on the camera in the case of using the original distances of the DI. Second, bringing the camera as close as possible to the lens *L*_1_, increases the amount of scattered rays that are captured by the camera and contributes to the resolution enhancement.

The SRI-COACH system is calibrated by illuminating the system from a point object located on the optical axis, where the corresponding intensity pattern, the PSH, is recorded by the camera. The object hologram is recorded by positioning the object at the same axial location of the point object. The object image is reconstructed by nonlinear cross-correlation of the object hologram with the PSH^21^.

### Synthesis of the phase mask

As mentioned above, the phase mask used in the SRI-CAOCH is the combination of the CPM and DL. The CPM is computed using a modified GSA^27,29^, whereas the DL with the refractive lens *L*_1_ are used to satisfy the Fourier relation between the CPM and the camera plane. The Fourier relation between the CPM and the camera plane is guaranteed if the imaging condition between the point object and the camera plane is fulfilled^4^.

**Figure 8:**
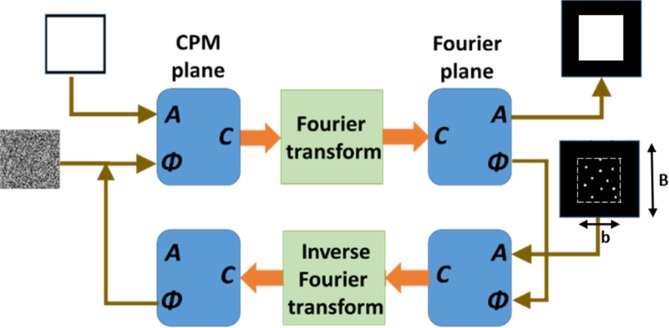
Modified Gerchberg-Saxton algorithm for synthesizing the CPMs.

As shown in Fig. 8, the iterative GSA algorithm^27,29^ is initialized with a random phase and a uniform amplitude on the CPM plane. The complex valued CPM is then Fourier transformed to the Fourier (camera) plane and the amplitude of the transformed CPM is replaced by the predefined randomly distributed dots distributed over a predefined area on the camera plane. The phase value of the transformed CPM is kept same. The algorithm is run, till the difference between the consecutive CPMs is negligible.

In the modified GSA^25^, the factors defining the PSH on the camera plane are the number of the dots randomly distributed and the size of the pre-defined area containing those dots. As a result of introducing the CPM, the effective NA of the system can be increased beyond the original NA. The increase of the NA depends on the scattering degree of the CPM and the focal length of the DL, as well as the position of the phase mask in the system. As seen in Fig. 7(b), the increase of the effective NA is caused by both the DL and the CPM. The light scattered by CPM is controlled by the scattering degree *σ* defined as *σ* = (*b*/B), where *b* is the constrained spectral bandwidth and *B* is the maximum spectral bandwidth, both are in the spectral domain of the CPM. The DL parameter controlling the scattered light is defined by its focal length *f*_*D*_.

The resolution enhancement is calculated next in the *σ*-limited region, in which NA < Min{*W*_*x*_*,W*_*y*_}/2*d*, where *W*_*x*_ is the width, *W*_*y*_ is the height of the CPM and *d* is the distance between the SLM and lens *L*_1_,. We assume *θ*_*σ*_ is the scattering angle of the CPM for a defined *σ*, such that the marginal ray from part of the phase mask with diameter *W*′ can enter the lens *L*_1_ due to the scattering. Then, the minimal resolved size of the system is $\delta x=1.22\lambda u/W{\prime}$, where *λ* is the central wavelength of the illumination and *u* is the distance between the point object and the SLM. For the product of the DL and the CPM, the DL forms a virtual image of the object at *v’* and the CPM further scatters the light into the input aperture of the imaging system (Fig. 7(b)). The lens equation for the DL is

1 $$\begin{array}{c}\frac{1}{{f}_{D}}=\frac{1}{v{\prime} }+\frac{1}{u}=\frac{2\,\tan ({\theta }_{f})}{W\text{'}}+\frac{1}{u}\\ {\theta }_{f}\simeq \frac{W{\prime} }{2}\left(\frac{1}{{f}_{D}}-\frac{1}{u}\right).\end{array}$$

The CPM scatters marginal rays traveling beyond the input aperture of the system into the input aperture of the imaging system. The trigonometric equation of the scattering is

2 $$\frac{W{\prime} -D}{2d}=\,\tan ({\theta }_{\sigma }+{\theta }_{f}),$$

where ${\theta }_{\sigma }=\sigma \lambda /2\Delta$ and Δ is the pixel size of the CPM. Using the small angle approximation, the diameter of the effective aperture is

3 $$W{\prime} =\frac{u{f}_{D}d}{{f}_{D}d-ud+u{f}_{D}}\left(\frac{D}{d}+\frac{\sigma \lambda }{\Delta }\right)$$

Hence, the minimal resolved size is $\delta x=1.22\lambda ({f}_{D}d-ud+u{f}_{D})/{f}_{D}d\left(\frac{D}{d}+\frac{\sigma \lambda }{\Delta }\right)$. For comparison with experimental results used in the section of 2D resolution-improved imaging (*u* = 49 cm, *f*_*D*_ = 23.3 cm, *d* = 11 cm and σ_o_ = 0.132), the effective diameter *W’* form Eq. (3) is 5.469 cm. The effective NA increment over the direct imaging is about 1.67 (5.469·60/4·49) which is with a close match to the experimental determined improvement of 1.6.

The second parameter in the GSA is the number of random dots which introduces the sparsity in the PSH. *N* defines the number of dots randomly taken in case of a particular scattering degree. Sparsity increases the SNR of the recorded holograms resulting in the decrease of noise in the reconstructed image. By decreasing the noise of the reconstructed image, unresolvable objects due to poor SNR becomes resolvable and hence the overall resolution performances of the system are further improved.

### Theoretical analysis

The goal of the following mathematical formalism is to obtain the expression of the PSH over the camera plane for any arbitrary CPM and to calculate the magnification of the imaging process. We choose to represent the CPM $C(\bar{r})$ as Fourier series of linear phases as follows,

4 $$C(\bar{r})=\exp [i\Phi (\bar{r})]=\mathop{\sum }\limits_{j=-\infty \,}^{\infty }{a}_{j}\,\exp (i2\pi {\bar{\upsilon }}_{j}\cdot \bar{r}),$$

where $\Phi (\bar{r})$ is the CPM phase synthesized using the modified GSA, $\bar{r}$ and ${\bar{\upsilon }}_{j}$ are the transverse location and the spatial frequency vectors, respectively. Assume the imaging condition is fulfilled between the point object and the camera plane, for CPM of a single linear phase, the image on the camera is the image of the point object, but shifted according to the parameters of the linear phase. This shift can be expressed by the delta function as the following,

5 $$I({\bar{r}}_{o})={|{a}_{j}\delta \left({\bar{r}}_{o}-\frac{\lambda v{u}_{e}{\bar{\upsilon }}_{j}}{4({u}_{e}+d)}\right)|}^{2},$$

where *u*_*e*_ = *uf*_*D*_*/*(*u* − *f*_*D*_), *u*, *d* and *v* are defined in Fig. 7(a) and ${\bar{r}}_{0}$ is the transverse location vector on the plane of the camera. For the entire linear phases composing the CPM, the intensity on the camera is as follows,

6 $$I({\bar{r}}_{o})={|\mathop{\sum }\limits_{j}^{\infty }{a}_{j}\delta \left({\bar{r}}_{o}-\frac{\lambda v{u}_{e}{\bar{\upsilon }}_{j}}{4({u}_{e}+d)}\right)|}^{2}.$$

The series of Eq. (6) represents the intensity of the Fourier transform of the CPM with the scaling operator of *ν*[4(*u*_*e*_ + *d*)*/λu*_*e*_*v*], where the scaling operator is defined by the equation *ν*[*α*]*f*(*x*) = *f*(*αx*). Therefore, the PSH, the intensity on the camera in response to an object point is,

7 $${I}_{PSH}({\bar{r}}_{0})={I}_{o}{|\nu \left[\frac{4({u}_{e}+d)}{\lambda v{u}_{e}}\right]{\mathfrak{F}}\{\exp [i\Phi (\bar{r})]\}|}^{2}$$

where *I*_*o*_ is a constant and ${\mathfrak{F}}$ is the operator of 2D Fourier transform. Apparently, the CPM can be synthesized using GSA, since the Fourier relation between the SLM and the camera planes is confirmed by Eq. (7). For an object point positioned at some arbitrary location on the input plane, the intensity on the camera plane is,

8 $$\begin{array}{ccc}{I}_{PSH}({\bar{r}}_{0};{\bar{r}}_{s}) & = & {I}_{o}{|\nu \left[\frac{4({u}_{e}+d)}{\lambda v{u}_{e}}\right]{\mathfrak{F}}\left\{\exp \left(\frac{i2\pi {\bar{r}}_{s}\cdot \bar{r}}{\lambda {u}_{e}}\right)\exp [i\Phi (\bar{r})]\right\}|}^{2}\\  & = & {I}_{PSH}\left({\bar{r}}_{0}-\frac{v}{4({u}_{e}+d)}{\bar{r}}_{s};0\right),\end{array}$$

The intensity on the camera plane is a shifted version {by ${\bar{r}}_{s}$ *v/*(4[*u*_*e*_ + *d*])} of the intensity response for a point object located on the optical axis $({\bar{r}}_{s}=0)$.

A 2D object illuminated by a spatially incoherent light and located at the same distance *u* from the SLM can be considered as a collection of *M* uncorrelated object points given as,

9 $$o({\bar{r}}_{s})=\mathop{\sum }\limits_{j}^{M}{a}_{j}\delta (\bar{r}-{\bar{r}}_{s,j}).$$

Since according to Eq. (8) the system is linear and space invariant, the intensity distribution for the 2D object on the camera plane is a sum of all the shifted point responses, given by,

10 $${I}_{OBJ}({\bar{r}}_{0})=\mathop{\sum }\limits_{j}^{M}{c}_{j}{I}_{PSH}\left({\bar{r}}_{0}-\frac{v}{4({u}_{e}+d)}{\bar{r}}_{s,j}\right),$$

### Reconstruction analysis

Reconstruction of object is done using nonlinear reconstruction (NLR) technique^21^. In the NLR technique, the magnitudes, $|{\hat{I}}_{OBJ}|$ and $|{\hat{I}}_{PSH}|$, are raised to the power of *o* and *r*, respectively, where ${\hat{I}}_{OBJ}$ and ${\hat{I}}_{PSH}$ are the Fourier transforms of *I*_*OBJ*_ and *I*_*PSH*_ given in Eqs. (7) and (10), respectively. In the NLR optimization procedure, only the spectral magnitudes of the object, and of the reconstructing function, are raised to the power of *o* and *r*, respectively, while the phase information remains intact. For an object of a point located at ${\bar{r}}_{s}$, the Fourier transform of the cross-correlation between the object and the PSH-based reconstructing function is,

11 $$\begin{array}{rcl}{I}_{REC} & = & {{\mathfrak{F}}}^{-1}\{{\mathfrak{F}}\{{I}_{OBJ}^{{\prime} }\otimes {I}_{PSH}^{{\prime} }\}\}={{\mathfrak{F}}}^{-1}\{{\hat{I}}_{OBJ}^{{\prime} }\cdot {\hat{I}}_{PSH}^{{\prime} \ast }\}\\  & = & {{\mathfrak{F}}}^{-1}\{{|{\hat{I}}_{PSH}|}^{o}\exp [i({\varphi }_{PSH}+2\pi {\bar{r}}_{s}\cdot {M}_{T}\cdot \bar{\upsilon })]{|{\hat{I}}_{PSH}|}^{r}\exp [-i{\varphi }_{PSH}]\}\\  & = & {{\mathfrak{F}}}^{-1}\{{|{\hat{I}}_{PSH}|}^{o}\exp [i2\pi {\bar{r}}_{s}\cdot {M}_{T}\cdot \bar{\upsilon }]{|{\hat{I}}_{PSH}|}^{r}\}=\delta ({\bar{r}}_{R}-{\bar{r}}_{s}\cdot {M}_{T}),\end{array}$$

where ${\bar{r}}_{R}$ is the transverse location vector on the reconstruction plane, ${\hat{I}}_{OBJ}^{{\prime} }$ and ${\hat{I}}_{PSH}^{{\prime} }$ are the Fourier transforms of ${I}_{OBJ}^{{\prime} }$ and ${I}_{PBH}^{{\prime} }$, respectively and the symbol $\otimes$ represents a two-dimensional correlation. ${I}_{OBJ}^{{\prime} }$ and ${I}_{PBH}^{{\prime} }$ are the patterns ${I}_{OBJ}$ and ${I}_{PSH}$, respectively, after the nonlinear operations described above. $\bar{\upsilon }$ is the spatial frequency vector and *M*_*T*_ is the overall transverse magnification of the imaging system given according to Eq. (8) by the ratio *M*_*T*_ = *v/*[4*(u*_*e*_ + *d)*].

In order to determine the optimal values of *o* and *r*, we varied both of them in the range of −1 and 1 $(-1\le o\le 1,-\,1\le r\le 1)$ with a predefined step of 0.1. Entropy was used as a blind figure-of-merit^21^ and the reconstruction result having the lowest entropy determined the optimal values of *o* and *r*. In order to calculate the entropy, firstly the reconstructed image *I*_*REC*_(*m,n*) was normalized as

12 $$\phi (k,l)=\frac{{I}_{REC}(k,l)}{\sum _{k}\sum _{l}{I}_{REC}(k,l)},$$

where $\phi (k,l)$ is the normalized intensity distribution function and *k,l* are the pixel coordinates of the image. The entropy is then calculated as

13 $$S(o,r)=-\,\sum _{k}\sum _{l}\phi (k,l)\log [\phi (k,l)].$$

The reconstruction results for the different values of the scattering degree and the dot number was analyzed by visibility and SNR values^27^. For the SNR calculation, the noise was calculated by averaging the background around the reconstructed object, whereas the signal was calculated by averaging over the reconstructed object. The visibility was calculated as $\nu =({I}_{max}-{I}_{min})/({I}_{max}+{I}_{min}),$ where *I*_*min*_ and *I*_*max*_ are the minimum and maximum intensities corresponding to the line profile, averaged along the grating of the reconstructed object. In order to determine the optimal dot number *N*_o_ and the scattering degree *σ*_*o*_, the product of visibility and SNR for every combination of *N* and *σ* was calculated as $\xi (N,\sigma )=\nu (N,\sigma )\cdot SNR(N,\sigma )$ and the highest value determines the optimal value.
